# Approaches for *in silico* finishing of microbial genome sequences

**DOI:** 10.1590/1678-4685-GMB-2016-0230

**Published:** 2017

**Authors:** Frederico Schmitt Kremer, Alan John Alexander McBride, Luciano da Silva Pinto

**Affiliations:** 1 Programa de Pós-Graduação em Biotecnologia (PPGB), Centro de Desenvolvimento Tecnológico, Universidade Federal de Pelotas, Pelotas, Brazil

**Keywords:** microbial genetics, molecular microbiology, genomics, microbiology, draft genomes

## Abstract

The introduction of next-generation sequencing (NGS) had a significant effect on the availability of genomic information, leading to an increase in the number of sequenced genomes from a large spectrum of organisms. Unfortunately, due to the limitations implied by the short-read sequencing platforms, most of these newly sequenced genomes remained as “drafts”, incomplete representations of the whole genetic content. The previous genome sequencing studies indicated that finishing a genome sequenced by NGS, even bacteria, may require additional sequencing to fill the gaps, making the entire process very expensive. As such, several *in silico* approaches have been developed to optimize the genome assemblies and facilitate the finishing process. The present review aims to explore some free (open source, in many cases) tools that are available to facilitate genome finishing.

## Introduction

The advent of second generation of sequencing platforms, usually referred to as Next Generation Sequencing (NGS) technologies, promoted an expressive growth in the availability of genomic data in public databases, mainly due to the drastic reduction in the cost-per-base (Chain *et al.*, 2009). Compared to the Sanger sequencing technique (Sanger *et al.*, 1977), NGS platforms, like Illumina HiSeq, IonTorrent PGM, Roche 454 FLX, and ABI SOLiD, are able to generate a significantly higher throughput, resulting in a high sequencing coverage. However, they typically have a lower accuracy (in terms of average Phred-score in the raw data) and, in most cases, can only generate short-length reads (*short-reads*) (Liu *et al.*, 2012). The term “short-read” is commonly used to refer to the data generated by platforms such as Illumina, IonTorrent and SOLiD, as the length of their reads usually range from 30 bp (eg: SOLiD) to ~120 bp (*e.g.*, Illumina HiSeq), which is smaller than the length usually obtained by Sanger sequencing (~1 kb), and by the PacBio and Oxford Nanopore platforms (commonly referred to as “long-reads”).

The higher throughtput obtained by NGS has stimulated the development of new algorithms and tools that are capable of dealing with a larger volume of data and generating genomic assemblies in a reasonable time. Traditional sequence assemblers, like Phrap (http://www.phrap.org/phredphrap/) and CAP3 (Huang and Madan, 1999), were replaced with new ones, such as Velvet (Zerbino and Birney, 2008), ABySS (Simpson *et al.*, 2009), Ray (Boisvert *et al.*, 2010), SPAdes (Bankevich *et al.*, 2012) and SOAPdenovo (Luo *et al.*, 2012), many of which were based on the *De Bruijn* graph algorithm (Pevzner *et al.*, 2001; Compeau *et al.*, 2011). A large number of these software offerings are capable of assembling data from different sequencing platforms, called “hybrid assembly”; however, all of them exhibit some limitations and, even after several corrections and optimization steps, the generation of a finished genome continues to represent a complex task (Alkan *et al.*, 2011).

Genome finishing is achieved by converting a set of contigs or scaffolds into complete sequences that represent the full genomic content of the organism without unknown regions (gaps) (Mardis *et al.*, 2002), and aims a “closed” and full representation of the chromosome organization. On the contrary, a draft genome may be composed of a set of a few or several contigs or scaffolds (Land *et al.*, 2015). Although useful for many studies, the unfinished and fragmented nature of draft genomes may difficult analysis on comparative genomics and structural genomics (Ricker *et al.*, 2012). In addition, some genes may be missed if located in a region without coverage (*e.g.*, edges of contigs/scaffolds), or due to assembly errors (*e.g.*, repetitive regions that are “collapsed” into a single one) (Klassen and Currie, 2012).

Genome finishing represents a relevant step to reduce data loss and lead to a more accurate representation of the genomics features of the organism of interest. The traditional way to close gapped genomes includes: (1) design primers based on the edge of adjacent contigs, (2) PCR amplification, (3) Sanger sequencing, and (4) local assembly, usually with (5) manual curation. As this process is very time consuming, new *in vitro* approaches were developed to speed it up, including multiplex PCR (Tettelin *et al.*, 1999), optical mapping (Latreille *et al.*, 2007), and hybrid sequencing and assembly (Ribeiro *et al.*, 2012). This, however, usually results in a drastic increase in the cost of the sequencing project. Therefore it is not surprising that from the 87,956 prokaryote genomes available in GenBank until December 2016 (GenBank release 217), only a small fraction (~6,586) is finished (http://www.ncbi.nlm.nih.gov/genbank/).

In the last years, the availability of third-generation sequencing technologies, such as PacBio SMRT and Oxford Nanopore, has also provided another way to achieve finished genomes (Ribeiro *et al.*, 2012). As these platforms usually generate reads with length > 10 kb, the assembly algorithms have to deal with less ambiguities and problematic regions (Koren and Phillippy, 2015). This makes the reconstruction of the chromosome sequences easier, but just like second generation technologies, both platforms have their own limitations. In the earlier versions of the PacBio SMRT platform, for example, it used to present a high error-rate, so it was recommended to correct part of these errors using short-reads data (*e.g.*, Illumina) (Koren *et al.*, 2012; Au *et al.*, 2012; Salmela and Rivals, 2014) before using its result for any analysis. Although this problem has been minimized with the improvements in the sequencing chemistry and base-calling process over the last years, the time required for the sequencing, the cost of each run, and the price of the equipment itself are still drawbacks, and these are some of reasons as to why it is not more frequently applied. In the case of Oxford Nanopore, there is a limited number of tools that can be used to pre-process and analyze its data, and some types of errors (*e.g.*, under-representation of specific k-mers) are still recurrent (Deschamps *et al.*, 2016). Therefore, second-generation platforms are still the most popular ones for a wide variety of applications, and remain as the cheaper alternative to obtain genomic data.

Previous reviews that focused on genome assembly and whole-genome sequencing analysis have already described some of the *in silico* tools that have the potential to improve a genome assembly without the need for experimental data. Edwards and Holt (2013), Dark (2013) and Vincent *et al.* (2016) have provided very comprehensive descriptions of some of the main steps of data analysis in microbial genomes sequenced by next-generation technologies, including *de novo* assembly, reference-based contig ordering, genome annotation and comparative genomics, and these are useful starting points for those that are new to microbial genomics with NGS. Nagarajan *et al.* (2010) have also made some useful considerations regarding genome assembly and presented some methodologies for genome finishing, but limited to a small set of approaches that they have developed to improve assemblies. Ramos *et al.* (2013) exemplified some assembly and post-assembly methods for genomes sequenced with IonTorrent platforms. Finally, Hunt *et al.* (2014) have also made a complete and accurate benchmark analysis of different tools for assembly scaffolding based on paired-end/mate-pair data. However, although very instructive, these reviews provide descriptions of some specific procedures, but do not give an in-depth view of the different finishing strategies, nor of the algorithms that are used in background by different tools from each category. Therefore, a more complete description of these tools may be useful, especially for those researchers that are starting to work with microbial genome analysis and want to achieve better assemblies without relying on re-sequencing or manual gap-closing.

**Figure 1:**
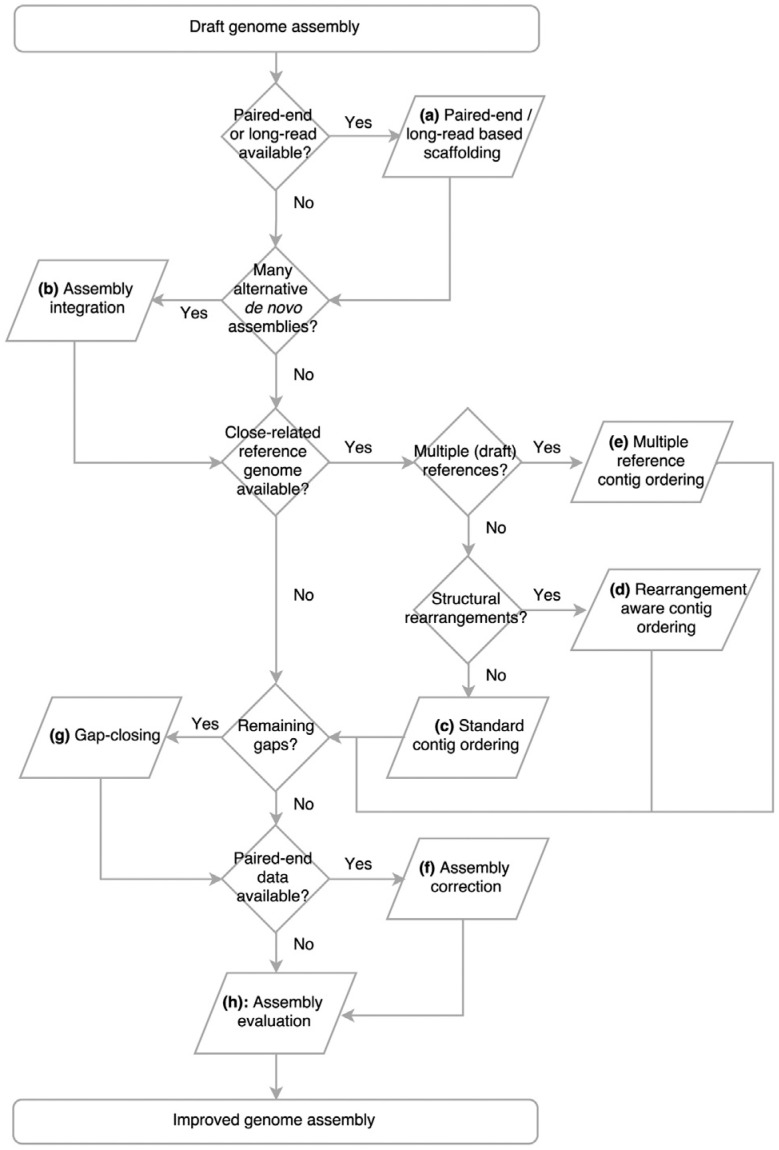
A flowchart demonstrating how and when the different genome finishing approaches can be combined according to the data that is available for the user. (a) *Scaffolding using paired-end reads or long-reads*, which is directly dependent on the way the genome was sequencing (platform, library), and sometimes performed as part of the *de novo* assembly process. (b) *Assembly integration*, which consists in the combination of different *de novo* assemblies and generation of a consensus/extended assembly. Some programs use only the assemblies as input, while others use also the sequencing reads. (c) The *standard contig-ordering* approach based on a single reference genome, which consists in the identification of synteny blocks that guide the orientation of the contigs in the draft genome, without taking into count the occurrence of genome inversions other rearrangements. (d) The *rearrangement-aware contig-ordering*, that identifies potential sites of inversion and translocations based on signatures on the alignment against the reference genome. (e) *The multiple-reference contig ordering*, that may be more appropriate in those cases where there is no finished reference genome, but there is a relatively high number of close-related drafts, or when there are no apparent closest reference to be used. (f) *Assembly correction*, which consists in the removing of short misassemblies, including base-substitutions and short insertions and deletions. (g) *Gap-closing*, which consists in the joining of adjacent contigs that used to be spaced by a gap. (h) *Assembly evaluation*, which may provide help to access the reliability of the assembly.

The present review aims at discussing some of the categories of software that may be applied in the process of assembly finishing, especially in the context of microbial genome sequencing projects. A particular focus is placed on those that do not require additional experimental and/or new sequencing data. The review intentionally focuses on tools that are freely available, at least for academic use, and omits proprietary software, like the CLC Genomics Workbench (http://www.clcbio.com/), for example. This was not due to anticipated differences in the performance or quality of the results, but to adhere to the original intention: an overview of tools that could be used by most researchers. As different approaches have been developed to improve genomic assemblies, the description of these programs was divided into four main categories: *scaffolding* (placement of contigs into larger sequences by using experimental data or information for closely-related genomes, and joining them by gap regions), *assembly integration* (generation of a consensus assembly using multiple assemblies for the same genome), *gap closing* (solving gaps by identifying their correct sequence), *error correction* (removal of misidentified bases or misassembled regions) and *assembly evaluation* (quantification of the reliability of a genome assembly and identification of its erroneous regions) (Table 1). These different categories of programs can be combined, according to the type of data that is available (*e.g.*, sequencing platform and library that was used), and may help to reduce the fragmentation and improve the reliability of a genome assembly. Based on the categories of software that are discussed in the present review we have created the flowchart showed in Figure 1, which may of help in choosing the most appropriate approach for genome finishing, depending on the type of the data that is available. Examples of genome projects that used some of the tools discussed in the present review are shown in Supplementary material (Table S1).

**Table 1:** Overview of the tools described in the present review

Category	Tool	Main features	Dependences*	Reference	Download link / webserver
Scaffolding	ABySS	– Paired-end scaffolding. – Scaffolding feature already integrated in the ABySS *de novo* assembly pipeline. – Uses the estimated distances generated by the program DistanceEst (from the same package) as input. – Allows the scaffolding using long-reads, such as those generated by PacBio and Oxford Nanopore platforms.	boost libraries: www.boost.org/ Open MPI: http://www.open-mpi.org sparse-hash library: http://goog-sparsehash.sourceforge.net/	(Simpson *et al.*, 2009)	http://www.bcgsc.ca/platform/bioinfo/software/abyss
Scaffolding	Bambus 2	– Paired-end scaffolding. – Can be easily integrated with assembly projects that are built on top of the AMOS package. – Supports the scaffolding of metagenomes. – Requires experience with the AMOS package and its data formats.	AMOS package (Treangen *et al.*, 2011): http://amos.sourceforge.net/	(Koren *et al.*, 2011)	https://sourceforge.net/projects/amos/
Scaffolding	MIP	– Paired-end scaffolding. – Supports both paired-end and mate-pair (long range) reads.	lpsolve library: http://sourceforge.net/projects/lpsolve/ lemon library: http://lemon.cs.elte.hu/	(Salmela *et al.*, 2011)	https://www.cs.helsinki.fi/u/lmsalmel/mip-scaffolder/
Scaffolding	OPERA	– Paired-end scaffolding. – Identifies potential spurious connections caused by chimeric reads and repetitive genomics elements that may affect the reliability of the scaffolding. – Contigs identified as misassembled may be used in the construction of more than one scaffold, but sometimes it may lead to new assembly errors.	BWA (Li and Durbin 2009): http://bio-bwa.sourceforge.net/ Bowtie (Langmead *et al.*, 2009): http://bowtie-bio.sourceforge.net/ Samtools (Li *et al.*, 2009): http://samtools.sourceforge.net/	(Gao *et al.*, 2011)	https://sourceforge.net/projects/operasf
Scaffolding	SCARPA	– Paired-end scaffolding. – Only uses for scaffolding those contigs with length greater than the N50 of the assembly. – Allows multiple libraries to be used in the same scaffolding project.	None	(Donmez and Brudno, 2013)	http://compbio.cs.toronto.edu/hapsembler/scarpa.html
Scaffolding	SGA	– Paired-end scaffolding. – Scaffolding feature already integrated in the SGA assembly pipeline, which is optimized for Illumina data and large genomes. – Uses the estimated distances generated by the program DistanceEst (from the package ABySS) as input, along with the read mapping file in .BAM format. – Allows multiple libraries to be used in the same scaffolding project.	Bamtools (Barnett *et al.*, 2011): https://github.com/pezmaster31/bamtools BWA (Li and Durbin, 2009): http://bio-bwa.sourceforge.net/ Samtools (Li *et al.*, 2009): http://samtools.sourceforge.net/ Sparse-hash library: http://goog-sparsehash.sourceforge.net/	(Simpson and Durbin, 2012)	https://github.com/jts/sga
Scaffolding	SOPRA	– Paired-end scaffolding. – Developed to improve the assemblies generated by Velvet and SSAKE, and required the .AFG files. – Supports data from early Illumina and ABI SOLiD platforms, including paired-end and mate-pair reads. – Is not fully automated, so it is necessary to run different scripts for each step of the scaffolding.	None	(Dayarian *et al*., 2010)	http://www.physics.rutgers.edu/~anirvans/SOPRA/
Scaffolding	SSPACE	– Paired-end scaffolding. – Trims the edge of the contigs as they are more suitable to assembly errors. – Requires information about the paired-end library, including mean size of the insert, standard deviation and the relative orientation of the mates.	None	(Boetzer *et al.*, 2011)	http://www.baseclear.com/genomics/bioinformatics/basetools/
Scaffolding	SSPACE-LongRead	– Paired-end scaffolding. – Allows the scaffolding using long-reads, such as those generated by PacBio and Oxford Nanopore platforms.	None	(Boetzer and Pirovano, 2014)	http://www.baseclear.com/genomics/bioinformatics/basetools/
Scaffolding	MUMmer	– Single reference-based scaffolding. – The result of the alignment must be post-processed to obtain the scaffolds.		(Kurtz *et al.*, 2004)	http://mummer.sourceforge.net/
Scaffolding	ABACAS	– Single reference-based scaffolding. – Useful when the reference and the target genome are closely-related, and the genome to be scaffolded is not larger than the reference genome. – Not optimized for bacteria with two or more replicons/chromosomes (ex: *Leptospira* genus). – Allows the design of primers for gap-closing.	MUMmer (Kurtz *et al.*, 2004): http://mummer.sourceforge.net/ Primer3 (Koressaar and Remm, 2007; Untergasser *et al.*, 2012): http://primer3.ut.ee/	(Assefa *et al.*, 2009)	http://abacas.sourceforge.net/
Scaffolding	CONTIGuator	– Single reference-based scaffolding. – Useful when the target genome is composed by more than one chromosome / replicon. – Allows a more sensitive identification of syntenic regions, if compared to ABACAS, as it applies a BLAST search after MUMmmer.	ABACAS (Assefa *et al.*, 2009): http://abacas.sourceforge.net/ BioPython (Python package): http://biopython.org/ BLAST+ (Altschul *et al.*, 1990; Camacho *et al.*, 2009): ftp://ftp.ncbi.nlm.nih.gov/blast/ MUMmer (Kurtz *et al.*, 2004): http://mummer.sourceforge.net/ Primer3 (Koressaar and Remm, 2007; Untergasser *et al.*, 2012): http://primer3.ut.ee/	(Galardini *et al.*, 2011)	http://contiguator.sourceforge.net/
Scaffolding	Mauve	– Single reference- based scaffolding. – Can be used both through a commandline interface (CLI) and a graphical user interface (GUI). – Allows the identification of genomic inversions and translocations. – Not optimized for bacteria with two or more replicons/chromosomes.	Java: https://www.java.com/	(Darling *et al*., 2004; Rissman *et al.*, 2009)	http://darlinglab.org/mauve/mauve.html
Scaffolding	FillScaffolds	– Single reference- based scaffolding. – Not optimized for bacteria with two or more replicons/chromosomes. – Results may require post-processing to reconstruct the sequence of the scaffold.	Java: https://www.java.com/	(Muñoz *et al.*, 2010)	Supplementary data of Muñoz *et al.* (2010). http://dx.doi.org/10.1186/1471-2105-11-304
Scaffolding	SIS	– Single reference-based scaffolding. – Allows the identification of genomic inversions. – Not optimized for bacteria with two or more replicons/chromosomes.	MUMmer (Kurtz *et al*., 2004): http://mummer.sourceforge.net/	(Dias *et al*., 2012)	http://marte.ic.unicamp.br:8747.
Scaffolding	CAR	– Single reference-based scaffolding. – Allows the identification of genomic inversions and translocations. – Also available as a webserver. – Not optimized for bacteria with two or more replicons/chromosomes.	MUMmer (Kurtz *et al*., 2004): http://mummer.sourceforge.net/ PHP: https://php.net/	(Lu *et al.*, 2014)	http://genome.cs.nthu.edu.tw/CAR/
Scaffolding	RACA	– Multiple reference-based scaffolding. – Optimized for large genomes and with multiple chromosomes. – Can also use paired-end data.	None	(Kim *et al.*, 2013):	http://bioen-compbio.bioen.illinois.edu/RACA/
Scaffolding	Ragout	– Multiple reference-based scaffolding. – Uses phylogenetic information to identify the most probable orientation of the contigs / scaffolds.	Networkx (Python package): http://networkx.github.io/ Newick (Python package): http://www.daimi.au.dk/~mailund/newick.html Sibelia: http://github.com/bioinf/Sibelia	(Kolmogorov *et al.*, 2014)	https://github.com/fenderglass/Ragout
Scaffolding	MeDuSa	– Multiple reference-based scaffolding. – Accepts both finished and draft genomes as reference.	BioPython (Python package): http://biopython.org/ Java: https://www.java.com/ MUMmer (Kurtz *et al*., 2004): http://mummer.sourceforge.net/	(Bosi *et al.*, 2015)	https://github.com/combogenomics/medusa
Assembly integration	Minimus	– Can be easily integrated with assembly projects that are built on top of the AMOS package. – Requires experience with the AMOS package and its data formats.	AMOS package (Treangen *et al.*, 2011): http://amos.sourceforge.net/	(Sommer *et al.*, 2007)	https://sourceforge.net/projects/amos/
Assembly integration	Reconciliator	– Corrects the misassembled regions in a target assembly by comparing to an alternative assembly for the same genome. – Identifies repetitive regions that suffered compressions or expansions.	MUMmer (Kurtz *et al.*, 2004): http://mummer.sourceforge.net/	(Zimin *et al.*, 2008)	http://www.genome.umd.edu/
Assembly integration	MAIA	– Allows the integration of two or more assemblies. – Accepts reference genome to perform scaffolding, what is useful for those contigs without correspondence in the other assemblies.	Matlab: https://www.mathworks.com/ MUMmer: http://mummer.sourceforge.net/ GAIMC (Matlab toolbox): http://github.com/dgleich/gaimc	(Nijkamp *et al.*, 2010)	http://bioinformatics.tudelft.nl
Assembly integration	CISA	– Allows the integration of three or more assemblies. – Corrects misassembled regions and compressed / expanded repeated regions.	BLAST+ (Altschul *et al*., 1990; Camacho *et al.*, 2009): ftp://ftp.ncbi.nlm.nih.gov/blast/ MUMmer (Kurtz *et al.*, 2004): http://mummer.sourceforge.net/	(Lin and Liao, 2013)	http://sb.nhri.org.tw/CISA/
Assembly integration	GAA	– Uses the alignment between the different contigs in the set of assemblies to generate an assembly graph, which is explored to identify to minimal set of independent paths.	BLAT (Kent, 2002): https://genome.ucsc.edu/ GSMapper: http://454.com/	(Yao *et al.*, 2012)	http://sourceforge.net/projects/gaa-wugi/
Assembly integration	Mix	– Generate an extension graph that represents the connection between the contigs. – Filters the alignment to reduce the ambiguities caused by repetitive sequences.	Networkx (Python package): http://networkx.lanl.gov/ BioPython (Python package): http://biopython.org/ MUMmer(Kurtz *et al.*, 2004): http://mummer.sourceforge.net/	(Soueidan *et al.*, 2013)	https://github.com/cbib/MIX
Assembly integration	GAM / GAM-NGS	– Requires the read files to perform the assembly integration. – One of the assemblies to be merged is defined as “master”, while the others are defined as “slaves”. – Allows the identification of misassembled regions in the master, which are corrected before the generation of the final assembly.	cmake: https://cmake.org/ zlib library: http://www.zlib.net/ boost libraries: www.boost.org/ sparse-hash library: http://goog-sparsehash.sourceforge.net/	(Casagrande *et al.*, 2009; Vicedomini *et al.*, 2013)	https://github.com/vice87/gam-ngs
Assembly integration	Zorro	– Requires the read files to perform the assembly integration. – Remaps the reads back to the contigs and identifies misassembled and repetitive regions based on the coverage. – Splits the misassembled contigs and performs the assembly integration using Minimus.	AMOS (Treangen *et al.*, 2011): http://amos.sourceforge.net/ BioPerl (Perl module): http://bioperl.org Bowtie (Langmead *et al.*, 2009): http://bowtie-bio.sourceforge.net/ MUMmer (Kurtz *et al.*, 2004): http://mummer.sourceforge.net/	(Argueso *et al.*, 2009)	http://lge.ibi.unicamp.br/zorro/
Gap closing	GapCloser	– Gap-closing feature already integrated in the SOAPdenovo *de novo* assembly pipeline – Performs a local reassembly in the gap region using the reads located in the edges of the surrounding contigs.	None	(Li *et al.*, 2010)	http://soap.genomics.org.cn/
Gap closing	IMAGE	– Iteratively performs a remapping of the reads to the contigs, followed by the selection of those that overlap the gap region and a local reassembly.	None	(Tsai *et al.*, 2010)	https://sourceforge.net/projects/image2
Gap closing	GapFiller	– Iteratively performs a remapping of the reads to the contigs, followed by the selection of those that overlap the gap region and a local reassembly. – Requires information about the paired-end library, including mean size of the insert, its standard deviation and the relative orientation of the mates.	None	(Boetzer and Pirovano, 2012)	http://www.baseclear.com/genomics/bioinformatics/basetools
Gap closing	Enly	– Iteratively performs a remapping of the reads to the contigs, followed by the selection of those that overlap the gap region and a local reassembly. – If a reference genome is provided, a new scaffolding step can be performed to improve the assembly.	BioPython (Python package): http://biopython.org/ BLAST and BLAST+ (Altschul *et al.*, 1990; Camacho *et al*., 2009): ftp://ftp.ncbi.nlm.nih.gov/blast/ Cdbfasta/cdbyank: http://compbio.dfci.harvard.edu/tgi/software/ EMBOSS: http://emboss.sourceforge.net/ Minimo assembler (Treangen *et al.*, 2011): http://amos.sourceforge.net/ MUMmer (Kurtz *et al.*, 2004): http://mummer.sourceforge.net/ Phrap: http://www.phrap.org/phredphrapconsed.html	(Fondi *et al*., 2014)	http://enly.sourceforge.net/
Gap closing	FGAP	– Uses alternative assemblies of the target genome to identify regions that overlap the gap.	Matlab: https://www.mathworks.com/	(Piro *et al.*, 2014)	http://www.bioinfo.ufpr.br/fgap/
Gap closing	Sealer	– Performs a local re-assembly of the gap regions using different settings of k-mer, what may help in the solving of regions with repetitive sequences.	boost libraries: www.boost.org/ sparse-hash library: http://goog-sparsehash.sourceforge.net/ Open MPI: http://www.open-mpi.org	(Paulino *et al*., 2015)	https://github.com/bcgsc/abyss/tree/sealer-release
Gap closing	GMCLoser	– May use both paired-end reads and alternative assemblies to perform the gap-closing. – Applies a likelihood analysis to avoid the effect of misassemblies in the alternative assemblies.	MUMmer (Kurtz *et al.* 2004): http://mummer.sourceforge.net/ BLAST+ (Altschul *et al*., 1990; Camacho *et al*., 2009): ftp://ftp.ncbi.nlm.nih.gov/blast/ Bowtie (Langmead *et al*., 2009): http://bowtie-bio.sourceforge.net/ YASS (Noé and Kucherov, 2005): http://bioinfo.lifl.fr/yass	(Kosugi *et al.*, 2015)	https://sourceforge.net/projects/gmcloser/
Gap closing	MapRepeat	– Performs a reference-based scaffolding using a closely-related genome provided by the user. – Uses a reference-guided assembly to perform the gap-closing process.	BLAST+ (Altschul *et al.*, 1990; Camacho *et al*., 2009): ftp://ftp.ncbi.nlm.nih.gov/blast/ BioPython (Python package): http://biopython.org/ MIRA: http://mira-assembler.sourceforge.net MUMmer (Kurtz *et al*., 2004): http://mummer.sourceforge.net/	(Mariano *et al*., 2015)	http://github.com/dcbmariano/maprepeat
Gap closing	GapBlaster	– Allows a manual gap-closing using an alternative assembly of the target genome.	BLAST and BLAST+ (Altschul *et al*., 1990; Camacho *et al.*, 2009): ftp://ftp.ncbi.nlm.nih.gov/blast/ MUMmer (Kurtz *et al*., 2004): http://mummer.sourceforge.net/	(de Sá *et al*., 2016)	https://sourceforge.net/projects/gapblaster2015/
Assembly evaluation	REAPR	– Calculates the accuracy of the assembly based on the coverage after remapping the reads back to the scaffolds. – Misassembled regions can be identified as they usually present a discrepant coverage. – A new set of scaffolds is generated by splitting the regions identified as misassembled.	File::Basename, File::Copy, File::Spec, File::Spec::Link, Getopt::Long and List::Util (Perl modules): http://www.cpan.org/ R: https://www.r-project.org/	(Hunt *et al.*, 2013)	http://www.sanger.ac.uk/science/tools/reapr
Assembly evaluation	QUAST	– Calculate several assembly metrics, such as C+G%, N50 and L50. – Can be used to compare different assemblies for the same genome, and / or compare then to a reference genome.	boost libraries: www.boost.org/ Java: https://www.java.com/ Matplotlib (Python package): http://matplotlib.org Time::HiRes (Perl module): http://www.cpan.org/	(Gurevich *et al*., 2013)	http://bioinf.spbau.ru/quast
Assembly evaluation	ALE	– Calculates the accuracy of the assembly based on the k-mers and C+G% distribution along the scaffolds. – Doesn't require a reference genome.	Matplotlib (Python package): http://matplotlib.org Mpmath (Python package): http://mpmath.org Numpy (Python package): http://www.numpy.org Pymix (Python package): http://www.pymix.org/pymix Setuptools (Python package): https://github.com/pypa/setuptools	(Clark *et al.*, 2013)	http://www.alescore.org
Assembly evaluation	CGAL	– Calculates the accuracy of the assembly based on the coverage after remapping the reads back to the scaffolds.	None	(Rahman and Pachter, 2013)	http://bio.math.berkeley.edu/cgal/
Assembly evaluation	GMvalue	– Aligns the assembly to a reference genome (or alternative assembly) to identify misassembled regions. – A new set of scaffolds is generated by splitting the regions identified as misassembled.	MUMmer (Kurtz *et al.*, 2004): http://mummer.sourceforge.net/ BLAST+ (Altschul *et al.*, 1990; Camacho *et al*., 2009): ftp://ftp.ncbi.nlm.nih.gov/blast/ Bowtie (Langmead *et al*., 2009): http://bowtie-bio.sourceforge.net/ YASS (Noé and Kucherov, 2005): http://bioinfo.lifl.fr/yass	(Kosugi *et al.*, 2015)	https://sourceforge.net/projects/gmcloser/
Assembly correction	iCORN	– Requires paired-end reads. – Interactively identifies and corrects short misassemblies, such as base-substitutions and short INDELs.	SNP-o-matic (Manske and Kwiatkowski, 2009): https://snpomatic.svn.sourceforge.net/svnroot/snpomatic SSAHA Pileup (Ning *et al*., 2001): ftp://ftp.sanger.ac.uk/pub/zn1/ssaha_pileup/	(Otto *et al.*, 2010)	http://icorn.sourceforge.net/
Assembly correction	SEQuel	– Requires paired-end reads. – Interactively identifies and corrects short misassemblies, such as base-substitutions and short INDELs. – Performs a local reassembly of the misassembled regions using information from k-mers and paired-end reads.	Java: https://www.java.com/ JGraphT (Java library): http://jgrapht.org/	(Ronen *et al*., 2012)	http://bix.ucsd.edu/SEQuel/
Assembly correction	GFinisher	– Doesn't require paired-end reads. – Integrates a reference-guided scaffolding step and gap-closing procedures, along with the assembly correction process. – Identifies misassembled regions based on the GC-Skew distribution.	Java: https://www.java.com/	(Guizelini *et al.*, 2016)	http://gfinisher.sourceforge.net/

* = Considering a computer running UNIX, Linux or Mac OS operating systems (OSs). As Make, sed, awk, GCC, Perl, Bash, Python and the GNU/Unix standard utility set are already included in most of the distributions / versions of these OSs, these programs were not listed as dependences.

### Scaffolding

By definition, a contig consists of a contiguous sequence has no unknown regions or assembly gaps (but may contain “N” that represent base-calling errors) (Staden, 1979). On the other hand, a scaffold consists of two or more contigs that have been joined according to some linkage information (*e.g.*, paired-end reads, genome maps) (Huson *et al.*, 2002). Paired-end or mate-pair libraries can be very useful in *de novo* genome assembly, and several tools use the relative position information to connect contigs into scaffolds (Hunt *et al.*, 2014). In a similar way, with the increase in the availability of genomic sequences from a wide variety of species, other scaffolding alternatives were developed to use one or multiple genomes as reference to order the contigs.

### Paired-end scaffolding

Most of the *de novo* genome assemblers usually integrate scaffolding steps after the contig constructions, although it is also possible to use third-party tools aiming a more reliable result. The A5 assembly pipeline (Tritt *et al.*, 2012), for example, uses *de novo* assembler IDBA (Peng *et al.*, 2010) to construct the contigs and SSPACE (Boetzer *et al.*, 2011) to generate scaffolds. The scaffolding with paired-end reads usually consist of the alignment of reads to the contigs, followed by the identification of connections between different contigs using the relative-orientation information and the estimated insert-size. ABySS (Simpson *et al.*, 2009), SOPRA (Dayarian *et al.*, 2010), SOAPdenovo (Li *et al.*, 2010), Bambus 2 (Koren *et al.*, 2011), MIP (Salmela *et al.*, 2011), Opera (Gao *et al.*, 2011), SSPACE (Boetzer *et al.*, 2011), SLIQ (Roy *et al.*, 2012), SGA (Simpson and Durbin 2012), SCARPA (Donmez and Brudno 2013), WiseScaffolder (Farrant *et al.*, 2015) and ScaffoldScaffolder (Bodily *et al.*, 2016) are examples of scaffolding tools based on paired-end information. More recently, the use of long-reads was also incorporated into scaffolding tools such as AHA (Bashir *et al.*, 2012) and SSPACE-LongRead (Boetzer and Pirovano, 2014).

ABySS (Simpson *et al.*, 2009): The program abyss-scaffold, which comes with the ABySS assembly package (Simpson *et al.*, 2009), uses the estimated mate-distance distribution in paired-end reads to connect contigs and generate scaffolds. The distance distribution can be calculated by DistanceEst, that is also part of the package, and is also used by other assembly pipelines, such as SGA (Simpson and Durbin, 2012). ABySS was developed to be used both with small and large genomes, and can be executed in a computer clustering by using the Message Passing Interface (MPI), this being useful also in case of high-coverage data and when dealing with multiple libraries. Like most scaffolding programs, abyss-scaffold can be used also in the scaffolding of contigs generated by third-party programs. Finally, ABySS also supports scaffolding with long-reads by using BWA-MEM for read alignment (Li and Durbin, 2009). The source code of the ABySS package is available at the address http://www.bcgsc.ca/platform/bioinfo/software/abyss, and is developed to work on the Linux operating system.

SOPRA (Dayarian *et al.*, 2010): This scaffolding tool was designed to improve assemblies generated by Velvet (Zerbino and Birney, 2008) and SSAKE (Warren *et al.*, 2007), and targets the earlier sequencing platforms from Illumina and ABI SOLiD. The program parses the read-placing file generated by these assemblers and extracts information of paired-end/mate-pair reads, that is used to calculate the mean distance between pairs and the correct orientation. Based on this file, SOPRA also infers the connections between contigs by searches of those pairs of reads where mates are in different contigs. The program is not fully automated, so each step of data processing must be executed by a different script before the main scaffolding process. Another drawback is the limited support for different *de novo* assemblers, as it requires read-placing files in AFG format, and this is only produced by a few assemblers nowadays (*e.g.*, Velvet, Ray and AMOS). SOPRA can be obtained from the website http://www.physics.rutgers.edu/~anirvans/SOPRA/.

Bambus 2 (Koren *et al.*, 2011): It is part of the AMOS package (Treangen *et al.*, 2011) and is both a genome and metagenome scaffolding tool and an updated version of the Sanger-based program Bambus targeting NGS data (Pop *et al.*, 2004). The program requires read-placing information to construct a contig-graph, and explore the graph to find consistent connections between the contigs. As Bambus2 can also be used to scaffold metagenomic assemblies, different from other programs, it considers the effect of DNA samples containing mixes of closely related organisms in the assembly processes and reduces the chance of fragmentation and miss-joining by analyzing the molecular variants. However, the use of Bambus 2 is not as simple as for other scaffolding tools, as it requires some experience with the AMOS tools to generate its input file and processes the outputs (Treangen *et al.*, 2011). The AMOS package can be obtained from the SourceForge repository: https://sourceforge.net/projects/amos/.

MIP (Salmela *et al.*, 2011): uses the concept of mixed integer programming to generate a set of scaffolds from a genome assembly and a set paired-ends/mate-pair reads. First, readaligner (Mäkinen *et al.*, 2010) is used to map the read-pairs back to the contigs. Then, the pairs are filtered to remove inconsistent connections, and the distances between the contigs are estimated based on the mean distance between the mates, which is calculated for each library used in the assembly. The connections in the generated scaffold graph have a minimum and maximum estimated length, derived from the library information. The MIP source code, along with usage instructions, is available at https://www.cs.helsinki.fi/u/lmsalmel/mip-scaffolder/.

Opera (Gao *et al.*, 2011): takes as inputs a collection of contigs and mapped reads and generates a scaffold graph based on the paired-end information. Frist, the program filters the connections between contigs to remove possible miss-joining errors caused by chimeric pairs. The graph is contracted, and the optimum orientation of the contigs inside the scaffolds is inferred by a dynamic programming algorithm that explores the search space. The algorithm can also infer the occurrence of repeated genomic regions, usually assembled into a single contig in case of short-reads. In this case, repeated regions are identified by comparing the coverage of the contigs to the mean coverage of the whole genome and selection those with value greater than 1.5 times the genomic mean. The identification of these regions allows a contig to be used in more than one scaffolds, which can provide a better assembly of repeated regions, but can also result in misassemblies. Opera can be obtained from its SourceForge repository https://sourceforge.net/projects/operasf/.

SGA (String Graph Assembler) (Simpson and Durbin 2012): is a *de novo* genome assembler developed for the memory-efficient assembly of small and large genomes by applying the method proposed by Myers (2005). As part of its assembly pipeline, SGA also provide a scaffolding tool that uses information from read alignment (in .BAM format), that can be generated by a wide variety of mapping tools (Li *et al.*, 2008; Langmead *et al.*, 2009; Lunter and Goodson, 2011), and estimated distance between mates, generated by DistanceEst, from the ABySS package, to connect contigs into scaffolds. SGA also supports scaffolding from multiple libraries, with different insert sizes, and was optimized to work with Illumina data. SGA is available from the GitHub repository https://github.com/jts/sga.

SCARPA (Donmez and Brudno, 2013): uses paired-end information to generate scaffolds, but takes into account that not only chimeric reads may be to responsible for inconsistent linkages between contigs but also misassembled sequences. It estimates the mean and standard deviation of the distance between the mates, but only uses information from those contigs with length greater than the assembly N50. The connections between the contigs are estimated based on the mate information and the calculated metrics, and if more than one library is provided, SCARPA process the scaffolding iteratively starting from the library with smaller insertion size. The program can be obtained from the URL http://compbio.cs.toronto.edu/hapsembler/scarpa.html.

SSPACE (Boetzer *et al.*, 2011) and SSPACE-LongRead (Boetzer and Pirovano, 2014): these scaffolding programs are currently distributed by BaseClear (http://www.baseclear.com), which also distributes the gap-closing program GapFiller (Boetzer and Pirovano, 2012). SSPACE requires information about paired-end library, including mean and standard deviation of distance between the mates and the expected orientation, whose values can be predicted with the script “estimate_insert_size.pl”, distributed along with the program. The user may choose between BWA (Li and Durbin, 2009) and Bowtie (Langmead *et al.*, 2009) for read mapping, the minimum number of connections to link two contigs, the number of bases that will be removed from the border of the contigs (as they usually contain errors), and the number of iterations. For SSPACE-LongRead, the target assembly is aligned to a collection of long-reads using BLASR (Chaisson and Tesler, 2012) and the alignments are filtered and refined to find the best orientation. Both SSPACE and SSPACE-LongRead can be requested from the BaseClear website http://www.baseclear.com/genomics/bioinformatics/basetools/.

Hunt *el al.* (2014) have performed an extensive comparison of the scaffolding tools and demonstrated that the quality of the resulting scaffolds is directly affected by the read-mapping program and the complexity of the genome. For the tested datasets, the best results were obtained by SGA, SOPRA and SSPACE, although all tested tools presented a certain percentage of miss-joined scaffolds in their outputs. Some scaffolding tools (*e.g.*, SGA) use pre-aligned reads as input, so the user is able to test and choose the read mapper. In this case, it is important to try different read mappers, taking into account that platform-specific bias and read quality may have a drastic effect on the quality of the alignment (Hatem *et al.*, 2013; Caboche *et al.*, 2014). When using mate-pair libraries (long-insert paired-end reads) it is also important to check if the scaffolder was designed to support it, or if it was just designed for standard, short-insert, paired-end reads. As mate-pairs may present a relatively high rate of “false mates”, special care may be taken when working with this type of data.

### Single reference-based scaffolding

**Figure 2:**
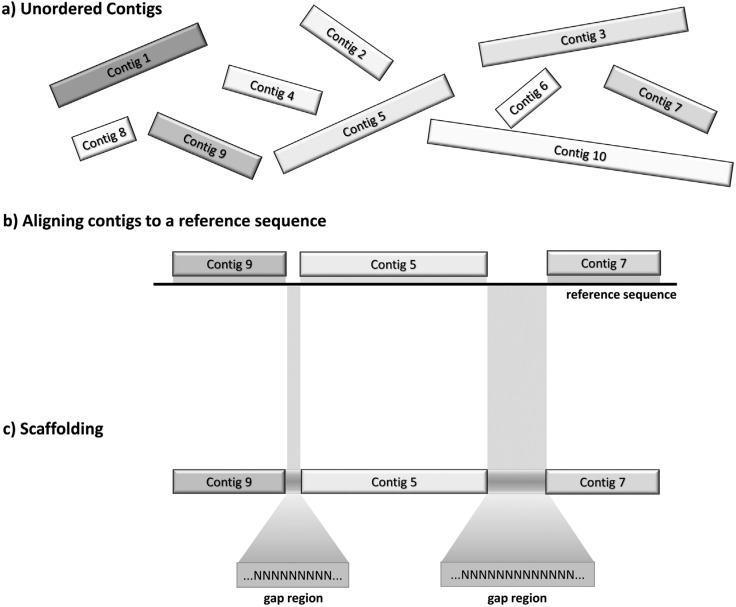
Reference-based contig ordering. (a) The program takes a set of contigs (or scaffolds) and (b) aligns these to a reference genome to identify the most probable relative orientation of the sequences in the draft genome. (c) Regions not covered by the contigs represent gaps and may be sequencing/assembling artifacts or natural deletions. Based on the relative position of each contig, a scaffold is created.

In many cases the pairing information is not enough to generate a reliable reconstruction of the genome's structure, or simply, the genome was not sequenced using paired-reads, but with single-end sequencing. In order to overcome this, some tools were developed to use a reference genome as a template to perform the contig ordering and relative positioning (Figure 2). Software like MUMmer (Kurtz *et al.*, 2004), ABACAS (Assefa *et al.*, 2009), CONTIGuator (Galardini *et al.*, 2011) and Mauve (Darling *et al.*, 2004; Rissman *et al.*, 2009) are able to identify the most probable orientation of the contigs, but may generate incorrect results in the case of genome inversions of translocations. On the other hand, SIS (Dias *et al.*, 2012), CAR (Lu *et al.*, 2014), and FillScaffolds (Muñoz *et al.*, 2010) consider the occurrence of changes in the genomic structure and take these phenomena into account during the analysis, generating a more accurate reconstruction. All these tools use information from a single genome as reference, however more recently, some tools, such as Ragout (Kolmogorov *et al.*, 2014) and MeDuSa (Bosi *et al.*, 2015), were developed to use information from multiple reference genomes, allowing an evolutionary-based inference of structural re-arrangements. These multiple reference-based contig ordering tools will be discussed in the next section.

MUMmer (Kurtz *et al.*, 2004): is a genome-scale sequence alignment tool which can be applied to perform the alignment of a set of contigs/scaffolds to a reference genome, allowing a wide variety of applications in genomic analysis and NGS data processing, including reference-guided scaffolding. The two main algorithms of the MUMmer package are NUCmer, which performs a standard DNA-DNA alignment, and PROmer, which performs an alignment of the six reading frames of both sequences (leading to a more sensitive result, especially in the case of highly divergent organisms). The package also includes other tools, such as delta-filter, that can be used to remove the ambiguities in the alignments and select those that are more relevant for the analysis. Many scaffolding tools, like ABACAS (Assefa *et al.*, 2009), CONTIGuator (Galardini *et al.*, 2011) and MeDuSa (Bosi *et al.*, 2015), are built on top of MUMmer and take advatange of its performance, but also add new features to improve the output. MUMmer itself does not provide the sequence of the scaffold, just the positions of the alignments. Therefore, it is necessary to perform a post-processing of the results to obtain the sequence of the scaffolds. MUMmer can be obtained from its SourceForge repository http://mummer.sourceforge.net/.

ABACAS (Algorithm-based Automatic Contiguation of Assembled Sequences) (Assefa *et al.*, 2009): can use NUCmer or PROmer from the MUMmer (Kurtz *et al.*, 2004) package to align the contigs against a reference genome. The regions that do not have an equivalent sequence in the contig set are filled with Ns, indicating gaps. ABACAS can also be used to design PCR primers to amplify the unknown regions by integrating Primer3 (Koressaar and Remm, 2007; Untergasser *et al.*, 2012). ABACAS can be obtained from its SourceForge repository http://abacas.sourceforge.net/, and as part of the PAGIT package (Swain *et al.*, 2012), available at http://www.sanger.ac.uk/science/tools/pagit.

CONTIGuator (Galardini *et al.*, 2011): uses ABACAS (Assefa *et al.*, 2009) to perform the contig ordering, but adds support to multiple references, which may be useful in the case of organisms that have more than one chromosome. BLAST (Altschul *et al.*, 1990; Camacho *et al.*, 2009) is used to align the contigs used as input with the reference sequences to identify the correct reference for each sequence. Then, ABACAS (Assefa *et al.*, 2009) is used, and its results are integrated with the BLAST alignment to generate a final assembly. CONTIGuator can be obtained from its SourceForge repository http://contiguator.sourceforge.net/, and is also available as a webserver http://combo.dbe.unifi.it/contiguator.

Mauve (Darling *et al.*, 2004; Rissman *et al.*, 2009): is an alignment tool that can handle and align multiple genomes and identify regions of high similarity called Locally Collinear Blocks (LCBs). One of the program's features, Mauve Contig Mover, performs contig ordering using the same algorithm (Rissman *et al.*, 2009). The program runs in an iterative mode, generating and optimizing the contig orientations based on the reference until no change is possible that can improve the model. A directory is generated for each iteration that contains inputs to visualize the genome in Mauve and a FASTA file with the sorted contigs. The Mauve aligner can be obtained from the URL http://darlinglab.org/mauve/mauve.html.

FillScaffolds (Muñoz *et al.*, 2010): analyzes the genomic distance between the contig set and a reference genome and generates an ordered sequence through identifying orthologous genes. It considers the effects of the evolutionary distance in the case of missing genes, and then uses the position of the orthologos present in the reference to order the contigs. The source code of FillScaffolds is available as a supplementary data of the Muñoz *et al.* (2010) paper at: http://bmcbioinformatics.biomedcentral.com/articles/10.1186/1471-2105-11-304.

SIS (Scaffolds from Inversion Signatures) (Dias *et al.*, 2012): takes as input a set of contigs in FASTA format and a coordinate file generated by NUCmer or PROmer (Kurtz *et al.*, 2004) after these contigs have been aligned with the reference sequence. Using the coordinates, the program searches for inversion signatures and generates a collection of orientations of the sequences that can be used to construct the scaffolds. The source code of SIS can be obtained from the URL http://marte.ic.unicamp.br:8747.

CAR (Contig Assembly using Rearrangements) (Lu *et al.*, 2014): uses NUCmer and PROmer in combination, unlike ABACAS (Assefa *et al.*, 2009) and SIS (Dias *et al.*, 2012), that use the result of only one. Based on the coordinates, CAR uses a block permutation model to generate the contig order by considering not only the effect of the genomic inversions, but also the occurrence of transpositions (Li *et al.*, 2013). CAR can be used from the webserver http://genome.cs.nthu.edu.tw/CAR/, where the source code is also available for download.

Considering the main algorithm of each program, it is important to keep in mind that the most appropriate tool for a given task will depend on the organism and the availability of the reference genomes. ABACAS (Assefa *et al.*, 2009) is very useful if the reference genome is larger than the target genome (considering the sum of the length of all contigs, and that all contigs have a homologous region in the reference), and the primer designing tools might be helpful in some cases; however, its sensibility decreases in cases of structural divergence. In such cases, other tools, like CONTIGuator (Galardini *et al.*, 2011) and Mauve (Darling *et al.*, 2004; Rissman *et al.*, 2009), may be more effective. Finally, SIS (Dias *et al.*, 2012) and CAR (Lu *et al.*, 2014) are indicated if the draft genome may present genomic inversions or transpositions. For most of the applications, these tools usually provide reliable results, especially for organisms that do not show a very variable genomic organization, and/or when there are enough finished genomes to properly choose the best reference. However, in some situations it may be necessary to evaluate different tools and references to check which one provides the best results. Finally, a single-reference may also lead to an “overfitted” ordering, especially when the reference is smaller, or in case of genomic inversions and translocations.

### Multiple reference scaffolding

Sometimes it is very difficult to identify the most appropriate reference genome to use for contig ordering, especially when structural rearrangements are common events in the genus/species of interests. Additionally, when using BLAST to identify the most “close-related” strain from a database of already finished genomes, it is not usual to find different strains as best hit for each contig. Finally, there are also those cases where no finished genome is available, but there are draft genomes of related strains. In these cases it would not be appropriate to use programs that take into account alignments against only one reference, so data from multiple organisms should be considered. The use of multi-references is relatively recent and another consequence of the advent of NGS, as there is more draft genomes than finished ones available in public databases. Examples of algorithms and programs that use this approach are RACA (Kim *et al.*, 2013), Ragout (Kolmogorov *et al.*, 2014) and MeDuSa (Bosi *et al.*, 2015).

RACA (Reference-Assisted Chromosome Assembly) (Kim *et al.*, 2013): uses local sequence alignment to identify co-linear synteny blocks. The synteny blocks are filtered using a length threshold, and based on the reference genomes, the probability of each synteny block adjacent to the others is calculated. This probability can also be combined with paired-end information to identify the most probable set of scaffold. The source code of RACA can be obtained from the URL http://bioen-compbio.bioen.illinois.edu/RACA/.

Ragout (Kolmogorov *et al.*, 2014): uses phylogenetic information and synteny blocks to order a set of contigs from a target genome using multiple genome references. First, Sibelia (Minkin *et al.*, 2013) is used to identify synteny blocks shared by the target and the reference sequences. Based on the synteny, the nucleotides of the genomes are represented as sequences of blocks, and the best “block orientation” is identified by a maximum parsimony, taking into account the block order in the reference genomes. The source code of Ragout can be obtained from its repository at GitHub https://github.com/fenderglass/Ragout.

MeDuSa (Multi-Draft based Scaffolder) (Bosi *et al.*, 2015): is a graph-based scaffolder that uses information from multiples references, which can be finished or draft genomes. The program uses NUCmer to alignment the target genomes to the references and construct a weighted graph based on the alignments were the nodes of the graph are connected by identifying those contigs that aligned to the same sequence in the references. In the next step, the orientation of each contig is assigned based on the alignment information and the most-probable ordered identified in the graph. The source code of MeDuSa can be obtained from the repository at GitHub https://github.com/combogenomics/medusa.

### Assembly integration

Different assemblers, or even the same assembler executed with different configurations, may produce different results. Minimum coverage, coverage cut-offs, minimum contig length, and k-mer size are examples of just some parameters that can affect the decisions of the assembler during the construction of the contigs (Baker, 2012). The way low-quality reads are treated, or how the correct paths in the assembly graph are constructed, is also different for each program. As different assemblies may present different representations of a given region in the genome, the construction of a “consensus assembly” can be an effective method of reducing assembly errors and generating an optimized set of contigs. This process, which is sometimes called “assembly reconciliation,” “assembly merging,” or “assembly integration,” can receive as input only a set of assemblies, as implemented in Minimus (Sommer *et al.*, 2007), Reconciliator (Zimin *et al.*, 2008), MAIA (Nijkamp *et al.*, 2010), CISA (Lin and Liao 2013), GAA (Yao *et al.*, 2012) and Mix (Soueidan *et al.*, 2013), or both a set of assemblies and the reads used for the assembly, as is the case with GAM-NGS (Vicedomini *et al.*, 2013) and Zorro (Argueso *et al.*, 2009).

Minimus (Sommer *et al.*, 2007): is an assembly tool from the AMOS package (Treangen *et al.*, 2011). Initially conceived to perform assembly of small genomes, it was posteriorly adapted for assembly integration. The main algorithm is based on the overlap-layout-consensus paradigm (Peltola *et al.*, 1984), which involves taking a set of sequences and performing several alignments to identify overlaps. The information provided by the alignments is used to construct a graph that is minimized by a combination of algorithms (Myers, 1995, 2005) to generate a final assembly. Minimus is available as part of the AMOS package, which can be obtained from the SourceForge repository https://sourceforge.net/projects/amos/.

Reconciliator (Zimin *et al.*, 2008): uses NUCmer, from the MUMmer package (Kurtz *et al.*, 2004), to identify assembly errors by comparing a template with a secondary assembly. With the alignments, the tool is able to identify the regions that have possibly suffered compression or expansion due to assembly errors in repetitive DNA sequences. The source code of Reconciliator is available from the URL http://www.genome.umd.edu/.

MAIA (Multiple Assembly Integrator) (Nijkamp *et al.*, 2010): uses the overlap-layout-consensus paradigm in a similar way to Minimus to construct a graph based on the overlaps identified by MUMmer (Kurtz *et al.*, 2004). The connections in the graph are used to construct a new assembly, and contigs that have no connection can be integrated with the assembly using a reference genome as a template. MAIA was implemented on top of the Matlab programming language and is available as a package for it that can be obtained from the URL http://bioinformatics.tudelft.nl.

GAA (Graph Accordance Assembly) (Yao *et al.*, 2012): is an assembly integration software that is based on a homonymous data structure. Taking a set of contigs as input, the tool uses BLAT (Kent, 2002) to generate alignments, identify overlaps and then generate a graph that represents the connections between the contigs. GAA is available from the SourceForge repository http://sourceforge.net/projects/gaa-wugi/.

CISA (Contig Integrator for Sequence Assembly) (Lin and Liao, 2013): uses a four-step algorithm to generate the merged assembly. First, a set of representative contigs is chosen from the individual assemblies. Assembly errors are identified by aligning all sets to one another, and any regions that are present in only one sequence are considered to be erroneous. In the event of errors, the contigs are broken in the incorrect portion into smaller sequences. The third step consists of generating several alignments using BLAST (Altschul *et al.*, 1990; Camacho *et al.*, 2009), and NUCmer (Kurtz *et al.*, 2004) to identify the optimal length of repetitive sequences. The information generated in the third step is used to construct the merged assembly in the final stage of the program. CISA can be obtained from the URL http://sb.nhri.org.tw/CISA/.

Mix (Soueidan *et al.*, 2013): uses alignments generated by NUCmer (Kurtz *et al.*, 2004) to generate an extension graph where the contigs are connected by their borders. The alignments are filtered to remove repetitive sequences, and this information is used to generate a graph. Finally, the algorithm parses the graph to identify the Maximal Independent Longest Path Set (MILPS) that represents the final assembly. The Mix source code is available at the GitHub repository https://github.com/cbib/MIX.

GAM (Genomic Assemblies Merger) (Casagrande *et al.*, 2009) and GAM-NGS (Genomic Assemblies Merger for Next Generation Sequencing) (Vicedomini *et al.*, 2013): GAM takes an assembly as a template, which is referred to as the “master”, and extends it using one or more sets of auxiliary assemblies (called “slaves”) by identifying blocks inside the sequences that were generated by the same reads. GAM was designed to run using contigs that are generated by Sanger sequencing, and it needs an AFG layout file that contains the position information of the reads used to construct the contigs. AFG files are only produced by a few NGS assemblers, like Velvet (Zerbino and Birney, 2008) and Ray (Boisvert *et al.*, 2010). A newer version, called GAM-NGS (Vicedomini *et al.*, 2013), was developed to work in the modern context of genome assembly and to avoid the requirement for a template file by using a read alignment file (BAM) (Li *et al.*, 2009) instead. Additionally, GAM-NGS can also identify misassemblies and perform corrections before generating the final assembly. The source code of GAM-NGS is available from the GitHub repository https://github.com/vice87/gam-ngs.

Zorro (Argueso *et al.*, 2009): Combines a preprocessing step based on the masking of repetitive DNA in the sequences, followed by the split of possible misassembled regions, and the assembly integration performed by Minimus (Sommer *et al.*, 2007). The misassembled regions are identified by using Bowtie (Langmead *et al.*, 2009) to remap the reads used for the assembly back to the contigs and by analyzing the coverage along the sequence. Zorro can be obtained from the URL http://lge.ibi.unicamp.br/zorro/.

The use of read alignment information, usually taken from BAM/SAM files, may help Assembly integrating tools to properly choose and identify bocks that are derived from the same genomic region, but, just like scaffolding, the quality of the process be directly affected by the sequencing platform and settings of the read mapper. Additionally, assembly integration with paired-end reads is usually much more time and memory consuming than scaffolding, so it is important to its important to check the system's availability before choose use this approach.

### Gap closing

**Figure 3:**
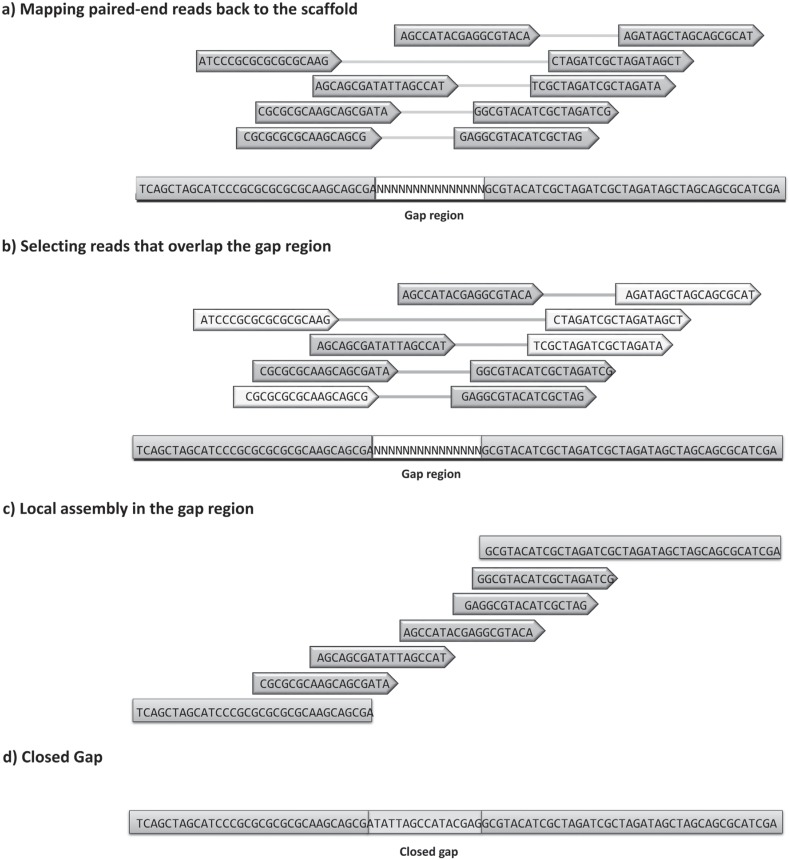
Example of a gap-closing approach using paired-end reads. (a) Taking as example a scaffold constituted by two contigs joined by an assembly gap (a run of `N's) by remapping the reads back to the contigs (b) it is possible to identify reads that have at least one of the mates in the gap region. Finally, (c) the reads identified inside the gap can be de novo assembled to fill the region, resulting in a (d) closed gap.

As described in the first section, different kinds of information may be used to generate scaffolds, like paired sequence, optical and/or genomics maps, and a reference genome. To improve the assembly, some algorithms were designed to close the gaps inside the scaffolds. One approach (Figure 3), which is used by tools like GapCloser (Li *et al.*, 2010; Luo *et al.*, 2012), IMAGE (Tsai *et al.*, 2010), GapFiller (Boetzer and Pirovano, 2012), Enly (Fondi *et al.*, 2014), MapRepeat (Mariano *et al.*, 2015) and Sealer (Paulino *et al.*, 2015), utilizes the information of single-end/paired-end reads to extend, and sometimes locally-reassemble, the contigs and close the gaps. Another approach, which is employed by FGAP (Piro *et al.*, 2014) and GapBlaster (de Sá *et al.*, 2016), uses an auxiliary set of contigs to find sequences that may fill the region based on local alignment. Finally, hybrid approaches, such as GMcloser (Kosugi *et al.*, 2015), may use a combination of paired-end data, alternative assemblies and long reads in the gap-closing process.

GapCloser (Li *et al.*, 2010): is part of the SOAPdenovo software package (Li *et al.*, 2010). This module uses the alignment algorithms from the package to realign the reads to the scaffolds. Based on the reads located near the gap, a local *de novo* assembly is performed constructed using a *De Bruijn* algorithm. In SOAPdenovo2 (Luo *et al.*, 2012), the GapCloser module was updated to deal with the possible errors caused by high divergent reads that might be used during the construction of the consensus sequence. GapCloser is distributed both as a stand-alone application or as part of the SOAPdenovo package, and can be obtained from the URL http://soap.genomics.org.cn/.

IMAGE (Iterative Mapping and Assembly for Gap Elimination) (Tsai *et al.*, 2010): takes as inputs a library of Illumina paired-end reads and a set of scaffolds and performs a remapping of the reads back to the sequences using SSAH2 (Ning *et al.*, 2001). Alternatively, the user can provide only the reads, and a *de novo* assembly is performed by Velvet (Zerbino and Birney, 2008) before the gap-closing step. The program maps the reads to the scaffolds and identifies those that are located at the border. Then, the reads pairs that have at least one of the mates on the border are selected, and a *de novo* assembly is performed by Velvet using the selected reads and contigs where the reads were mapped as inputs. After the assembly, the contigs are extended, and this increases the probability that the mates will align with the adjacent contigs or the gap will be filled after the assembly increases as more iterations are performed. At the end of the process, the contigs that were linked during the assembly can be ordered according to their previously known relative position. IMAGE https://sourceforge.net/projects/image2/ can be obtained from its SourceForge repository or as part of the PAGIT package www.sanger.ac.uk/science/tools/pagit.

GapFiller (Boetzer and Pirovano 2012): In a similar approach to that used by the previous tools, GapFiller performs a remapping of the paired-end reads back to the scaffolds. However, to run the program, it is necessary to know (or at less have an estimation of) the insert size and the orientation of the library used. If this information is not available, it is possible to calculate it using the script “estimate_insert_size.pl” that is distributed along with SSPACE. To perform the gap filling exercise, the program trims the contigs on both sides to reduce the possible assembly errors caused by low coverage and then applies an iterative process of read remapping and extension of the contigs. The alignment of the reads can be performed using BWA (Li and Durbin, 2009) or Bowtie (Langmead *et al.*, 2009) and the pairs that have one of the mates on the border of the contig and the other inside the gap region are identified. The contig extension uses a *k*-mer based assembly according to the reads found inside the gap. GapFiller, in the same way than SSPACE, is available for download at the BaseClear website http://www.baseclear.com/genomics/bioinformatics/basetools/.

Enly (Fondi *et al.*, 2014): is a simple gap-closing program that works by re-mapping reads, in FASTA format, back to a target assembly using BLAST (Altschul *et al.*, 1990; Camacho *et al.*, 2009), followed by a local assembly using Phrap (www.phrap.org/) or Minimo, from the AMOS package (Treangen *et al.*, 2011). If a reference genome is provided, a modified version of CONTIGuator (Galardini *et al.*, 2011) is used to order de target assembly and verify the accuracy of the gap-closing. The program can be obtained from the SourceForge repository http://enly.sourceforge.net/.

FGAP (Piro *et al.*, 2014): Unlike the other gap-filling tools, which utilize paired-end reads, FGAP's algorithm uses a supplementary set of long sequences, which can be a library of long-reads or an alternative assembly of the genome. After trimming the contigs, BLASTn, from the NCBI-BLAST+ package (Altschul *et al.*, 1990; Camacho *et al.*, 2009), is used to identify sequences in the supplementary set that overlaps the gap. FGAP can be download and used as a webserver from the URL http://www.bioinfo.ufpr.br/fgap/.

Sealer (Paulino *et al.*, 2015): The program was developed to be applied in large genomes, although can be applied in small prokaryote genomes as well. The main algorithm consists in the selection of nucleotides in the assembly that flank the gap-regions, followed by a local assembly by Konnector (Vandervalk *et al.*, 2014) using data from paired-end reads. Konnector, which takes the paired-end reads and generates pseudo-long reads by applying a combination of Bloom filter and *De Bruijn* graph, is distributed alongside with the recent versions of ABySS (Simpson *et al.*, 2009). Sealer evokes Konnector with different *k*-mers, what allows a more efficient closing of gaps caused by low coverage (usually closed by shorter *k*-mers) and repetitive elements (usually closed by longer *k*-mers). As it was developed aiming large genomes, Sealer tends to be more memory-efficient, but some steps of the program are executed serially, not in parallel, increasing the computation time. Sealer can be obtained from its GitHub repository https://github.com/bcgsc/abyss/tree/sealer-release.

GMCLoser (Kosugi *et al.*, 2015): combines information from paired-end reads and an alternative assembly, or long-reads, to fill gaps inside scaffolds. Paired-end reads are mapped to the target, and alternative assembly with Bowtie (Langmead *et al.*, 2009) and MUMmer (Kurtz *et al.*, 2004) is used to align the alternative assembly to the target assembly. To reduce the effect of misassembled regions that may be present in alternative assemblies, the program uses a likelihood approach to evaluate the contig joining by verifying the consistence based on the mate information. The likelihood algorithm available to evaluate assemblies can be accessed with the GMValue program, distributed alongside with GMCloser. The program can be obtained from its SourceForge repository https://sourceforge.net/projects/gmcloser/.

MapRepeat (Mariano *et al.*, 2015): is both a gap-closing and reference-guided scaffolding program. This software receives a genome assembly (in FASTA format), a reference genome (in Genbank format) and the sequencing reads (FASTA or FASTQ format). First, the assembly is ordered using the reference genome using CONTIGuator (Galardini *et al.*, 2011), and for each gap the adjacent contigs are aligned back to the reference using BLAST (Altschul *et al.*, 1990; Camacho *et al.*, 2009), aiming the identification of the region in the reference that is analogous to the missing region inside the gap in the target assembly. MIRA (www.chevreux.org) is used to align the reads to the reference and generate a consensus sequence, and the regions analogous to the gap are selected and used to close it. The source code of MapRepeat can be obtained from its GitHub repository http://github.com/dcbmariano/maprepeat.

GapBlaster (de Sá *et al.*, 2016): is a gap-closing programs that, in contrast with most of the other tools, allows a manual curation of the gaps in a draft assembly instead of providing an automated gap-closing algorithm. The user may choose between the legacy NCBI-BLAST package, NCBI-BLAST+ (Altschul *et al.*, 1990; Camacho *et al.*, 2009) or Nucmer (Kurtz *et al.*, 2004) to perform alignments between a set of contigs and the draft genome of interest, and then verify which alignments identified as flanking the gap regions may be considered for gap-closing.

The algorithms implemented in GapCloser (Li *et al.*, 2010; Luo *et al.*, 2012), IMAGE (Tsai *et al.*, 2010) and GapFiller (Boetzer and Pirovano, 2012) are based on paired-end information and are very useful for most of the genomes sequenced using Illumina or Roche 454 platforms. GapFiller requires not only the paired-end reads, but also an estimation of the size, of the insert (and its standard deviation) and the orientation of the read pairs (FR: forward-reverse, RF: reverse-forward, FF: forward-forward or RR: reverse-reverse), however, if such information is not available, it is possible to estimate it using the same script distributed along with SSPACE (Boetzer *et al.*, 2011).

In comparison to Gapfiller, Gapcloser, which also uses paired-end information for gap-closing, requires less information and may provide a more straightforward way to fill gaps. However, GapCloser also has its own limitations, including the lack of support for reads longer than 155 bp (nowadays, 2×250 bp paired-ends reads are very common for whole-genome sequencing, especially for microbial genome sequenced using Illumina MiSeq).

IMAGE is also very easy to be executed. It does not require prior knowledge about the library construction, and only needs the scaffolds, the reads and the number of iterations to be run. As it uses SMALT (based on hashes) instead of BWA or Bowtie (based on Burrows-Wheeler compression), the read-mapping also tends to be more sensitive and able to identify alignments, even if only part of the reads matches the sequence (*e.g.*, edges of contigs and gaps). However, as IMAGE does not handle insert size, it is unable to estimate the size of the gap, so the user must choose an arbitrary length after closing for those gaps that remained in the scaffolds. Although it is not problem when working with genomes assembled using only paired-end reads (with short-insert sizes), it may result in drawbacks if mate-pair reads where used for scaffolding, as long gaps would be confused with short ones.

More recently, FGAP (Piro *et al.*, 2014), GMcloser (Kosugi *et al.*, 2015) and GapBlaster (de Sá *et al.*, 2016) have provided another way to close gaps by using information for alternative assemblies, long reads or merged paired-end reads. In fact, GMcloser may also use information from paired-end reads, but by combining data from alternative assemblies and longs reads, it is possible to achieve a more reliable result. Sealer (Paulino *et al.*, 2015) also uses some of the advantages of long-reads, but by merging paired-end reads in artificial long one. Although it is not even close to PacBio or Oxford Nanopore reads, or not even to Sanger reads (~1 kb), this merging process may be helpful to solve some short unknown regions.

FGAP and GapBlaster are similar in the way they identify potential targets for gap-closing, but different in the sense that FGAP performs the gap-closing automatically whereas GapBlaster requires manual inspection. Although most of the tools are automated, the availability of tools for manual revision is also important, as some regions are very difficult to be assembled or corrected, and it is difficult to define generic rules to solve these situations.

### Error-correction and assembly evaluation

The combination of different approaches employed to assemble and finish a genome can result in some artifacts, caused by the limitations of each software and/or the platform used for sequencing. Tools like QUAST (Gurevich *et al.*, 2013), REAPR (Hunt *et al.*, 2013), ALE (Clark *et al.*, 2013) and GMvalue (Kosugi *et al.*, 2015) were developed to evaluate the accuracy of an assembly. Additionally, other softwares, like iCORN (Otto *et al.*, 2010) and SEQuel (Ronen *et al.*, 2012), are able to correct assembly errors, including insertions, deletions and base substitutions.

### Assembly evaluation

REAPR (Recognition of Errors in Assemblies using Paired Reads) (Hunt *et al.*, 2013): takes as input a FASTA file containing the scaffolds and a BAM file generated by the remapping of the reads used in the assembly. First, a coverage analysis is performed using SNP-o-matic (Manske and Kwiatkowski 2009) or SMALT (https://www.sanger.ac.uk/resources/software/smalt), for genomes that are smaller or bigger than 100 Mb, respectively. The software analyzes the assembly base-per-base and uses the information per position in a metric called *Fragment Coverage Distribution* (FCD), where the expected coverage distribution is compared to the observed values. The discrepant regions are treated as possible misassemblies and REAPR can generate a new set of scaffolds splitting the erroneous regions into separate sequences. REAPR can be obtained from the URL http://www.sanger.ac.uk/science/tools/reapr.

QUAST (QUality ASsessment Tool for genome assemblies) (Gurevich *et al.*, 2013): can be used to compare different assemblies of the same genome or to simply analyze an assembly. If more than one assembly is loaded, the program uses the MUMmer package to align the sequences and identify possible erroneous regions, count the number of aligned/unaligned bases, and also calculate, for each assembly, metrics like N50, L50, C+G% content, and other useful statistics. QUAST can be obtained from the URL http://bioinf.spbau.ru/quast, where it is also available through a webserver.

ALE (Assembly Likelihood Evaluation) (Clark *et al.*, 2013): uses a combination of statistical analysis that are mainly based on probability distribution and Bayesian inference to determine the accuracy of an assembly without requiring a reference genome. To evaluate the assembly, the program analyzes the *k*-mer distribution, C+G% and the relative orientation of the mates (paired-end reads) in the BAM file. ALE can be obtained from its website http://www.alescore.org.

CGAL (Computing Genome Assembly Likelihood) (Rahman and Pachter 2013): evaluates the accuracy of the assembly using a probability distribution analysis that takes into consideration the expected coverage with that obtained after the reads are remapped to the scaffolds. CGAL can be obtained from the URL http://bio.math.berkeley.edu/cgal/.

GMvalue (Kosugi *et al.*, 2015): is a program distributed along with the gap-closing program GMCloser, but can be used as a stand-alone application. The program uses NUCmer to align the assembly to a reference genome and identify misassemblies, such as insertions and deletions (INDELs). GMvalue can also generate “error-free” assemblies by splitting contigs in their erroneous regions. The program is distributed along with GMCloser and can be obtained from its SourceForge repository https://sourceforge.net/projects/gmcloser/.

When assembling a novel genome, the first metrics that are taken into account are the number of contigs, the length of the assembly and the N50. Although they can provide a good idea of how “contiguous” is the assembly, they do not measure its reliability, and may be easily distorted by inappropriate assembly procedures. Use of information from just one pair of reads to join two contigs into a scaffold, for example, may lead to an assembly error, even if the apparent fragmentation of the assembly is being reduced. If two regions are wrongly joined during the assembly, it may be misinterpreted as a natural biological event, and makes difficult further steps of assembly finishing.

### Assembly correction

**Figure 4:**
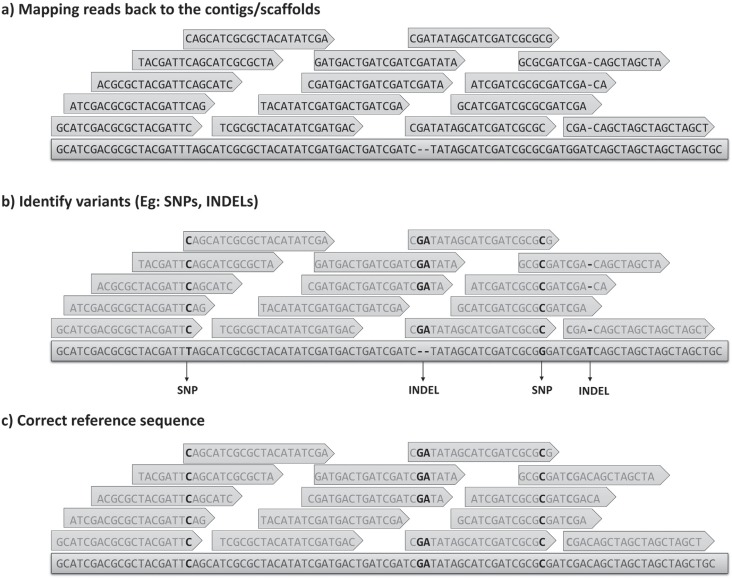
Example of a simplified assembly correction approach for base substitutions and insertion/deletion misassemblies. The process steps are (a) map the reads to the assembly, (b) identify variants (eg: SNPs and INDELs) in a similar way to the common variant calling analysis pipelines, and finally, (c) correct the regions in the assembly that show discrepancies. These steps may be reiterated several times until no further change be able to improve the assembly.

Assembly evaluation tools are very useful to identify structural inconsistences in a draft of apparently “finished” genome, but sometimes a manual revision and correction is inapplicable. Platform-specific errors, such as base-substitutions in Illumina data, or homopolymeric-sequence errors in IonTorrent data, may affect the annotation process, as some genes may be wrongly identified as frameshifted or mutated. As these types of errors may occur along the genome assembly, it is important to have automated tools to correct them automatically and reduce the chance of potential effects on the downstream analysis. ICORN and SEQuel are examples of programs that can be used to reduce these base-scale errors, and are based in the same principle that is used for variant calling analysis, consisting in the mapping of the reads against the sequence followed by the identification of those regions where there is a discrepancy, such as single-nucleotide polymorphism (SNPs) and Insertions and Deletions (INDELs) (Figure 4).

iCORN (Iterative Correction of Reference Nucleotides) (Otto *et al.*, 2010): is an automated pipeline for assembly correction. Using a paired-end library, the program performs the read remapping using SSAH (Ning *et al.*, 2001) and the variant calling and coverage analysis using SNP-o-matic (Manske and Kwiatkowski, 2009). The correction of the reference is followed by a new coverage analysis. If the correction promoted an improvement in the coverage, a new iteration commences, and the corrected sequence is used as the new reference; otherwise, the program stops and the last corrected sequence is returned as output. ICORN can be obtained from its SourceForge repository http://icorn.sourceforge.net/ and as part of the PAGIT package.

SEQuel (Ronen *et al.*, 2012): uses a modification of the widely used *De Bruijn* graphs called *positional De Bruijn graph*. Using the BWA's output (Li and Durbin, 2009), the main algorithms combine the *k*-mer information with the relative position and orientation information of the mates in the paired-end library to construct a graph that is used to generate a new set of corrected contigs. SEQuel can be obtained from the URL http://bix.ucsd.edu/SEQuel/.

Both iCORN and SEQuel can be used to reduce base-scale errors in genome sequences, such as single nucleotide substitutions errors and small artificial INDELs, but are not able to identify and correct genome-scale misassemblies, such as those identified by REAPR or GMValue. In fact, both REAPR and GMValue can be used to break misassembled genomes and generate a correct set of contigs, but they are not able to generate an improved assembly, in terms of contiguity, and new rounds of scaffolding and gap-closing would be necessary for this purpose. To address the problem of genome-scale assembly correction, Guizelini *et al.* (2016) have developed the tool GFinisher, which integrates the detection and correction of assembly errors with the reference-guided scaffolding and the gap-closing processes.

GFinisher (Guizelini *et al.*, 2016): is an assembly correction tool that also incorporates elements of assembly integration, reference-based scaffolding, and gap-closing in its internal pipeline. First, the program performs a reference-guided scaffolding using the module jContigSort (https://sourceforge.net/projects/jcontigsort/), which applies a HashMap-based algorithm using the *k*-mers from the draft genome and from a reference. Gaps between the contigs are then closed using jFGAP, a Java-implementation of the FGAP algorithm (Piro *et al.*, 2014), by taking information form alternative assemblies of the target genome. To identify misassembled regions, GFinisher uses an adaptation of the GC-skew metric (Lobry, 1996), which allows the analysis of fluctuations in the distribution of C and G along the sequence by using a sliding window. Points in the sequence where the GC-skew seems to be discrepant when compared to its context are considered as assembly errors, and are used to split the contigs. Then, the processes of reference-guided scaffolding, gap-closing and analysis of the GC-skew are repeated, and a final assembly is generated. At the end, all intermediary assemblies and the final one are analyzed by QUAST (Gurevich *et al.*, 2013). GFinisher can be obtained from its SourceForge repository http://gfinisher.sourceforge.net/.

While base-scale errors may affect the genome annotation by the presence of artificial frameshifts or non-synonymous mutations, the genome-scale misassemblies may lead to the erroneous identification of genome rearrangements or even the loss of relevant genes in the annotation, if they are located in the misassembled region. Therefore, the correction of base-scale errors and genome-scale misassemblies are crucial steps in the generation of high-quality finished assemblies.

## Conclusion

NGS platforms have provided new ways to obtain large amounts of genomics data and reduced expressively the cost of the sequencing process itself, but also brought new challenges, especially to the data management and analysis. In the case of genome sequencing projects, for example, depending on the organism and the sequencing platform, the size of the FASTQ files containing the raw reads may vary from less than 1 gigabyte (Gb) to thousands of Gbs. Although nowadays it is perfectly possible to perform the *de novo* assembly of a microbial genome using a desktop computer, or even a notebook, when the volume of data is relatively low, many NGS analysis still require high-performance computing infrastructures (*e.g.*, clusters, cloud computing, distributed systems) to be executed. In fact, even *de novo* genome assembly, which is in a certain way a well-established field, has been constantly renewed as more approaches are being developed to optimize memory usage (*e.g.*, string-graphs, compressed data structures) and the assembly process itself, especially due to the limitations implied by the short reads sequencers and, more recently, the relatively higher error-rate observed in third-generation sequencing platforms.

The development of new bioinformatics tools in the last decade was highly influenced by the new generation of sequencing platforms, and many software offerings were specifically designed to overcome the limitations of the NGS platforms and the challenges they have brought. The use of whole genome shotgun (WGS) sequencing became a common practice, and the amount of draft genomes available rose exponentially. However, the process by which a finished genome is generated, even in the case of bacteria, is still somewhat flawed and challenging.

The present review aimed to describe some tools that might be useful for improving genome assembly and facilitating the finishing process. For didactical purposes, the available tools were divided into four main groups. However, there are many other forms of improvements that can be used to optimize an assembly. Additionally, *in silico* tools can be very helpful and may reduce the need of re-sequencing. However, they also have some limitations, and it is important to know that their efficiency is directly dependent on the quality of the assembly and the reads.

As there are many aspects that may affect the assembly, from the sequence itself (*e.g.*, repetitive DNA, GC-rich or GC-poor) to technique artifacts (*e.g.*, platform bias, assembler errors), it is very difficult to create a straightforward method to turn millions of short-reads to close and error-free chromosome sequences. Therefore, when working with a newly sequenced organism, it may be useful to try different strategies for *de novo* assembly, assembly integration, scaffolding and gap-closing, and check how they affect the reliability of the genome assembly to choose the best parameters. Knowing the characteristics of the genomic structure of an organism, the sequencing platform and the library construction may also be very useful when choosing the tools, as some of them are designed and optimized to deal with certain specific error patterns.

Although not always applicable, there are many genome finishing tools that are also available as webservers, what may be very convenient for those researchers that are not fully confortable with Linux and commandline interfaces. Additionally, as most of these tools do not directly require the sequencing data or mapping files, the uploading and processing time is usually very fast.

Genome announcement papers that describe sequencing projects using the same technology may facilitate the choice, especially when the organism is closely related to the one you are interested to analyze. As new tools are constantly being developed and released, it may also be useful to check not only bibliography databases, such as PubMed, but also bioinformatics software repositories, such as OMICtools (https://omicstools.com), and web-based discussion forums, such as Biostars (https://www.biostarts.org) and SEQanswers (https://seqanswers.com), to keep updated about new programs and techniques.

The new generation of DNA sequencing platforms, such as PacBio RS II and Oxford Nanopore, will certainly guide the development of new bioinformatics tools and analysis protocols for the next years, and may provide an easier way to generate high-quality finished genomes. These sequencing platforms intended to produce reads that are much longer than the older second-generation platforms, like Illumina, IonTorrent, and SOLiD, and may overcome most of the limitations associated with short-reads. One of the most common issues associated with genome assembly that uses short-reads is that repeated DNA regions, like simple sequence repetitions (SSRs) that are longer than the read length, are considered computationally impossible to assemble “exactly”. However, today, the throughput of these new long-read sequencers is still low and has a relatively high error rate compared to alternative platforms. This indicates that many aspects of genome sequencing still require improvement. Additionally, sequencing by PacBio SMRT still very time consuming and expensive when compared to other platforms, such as IonTorrent PGM and Illumina MiSeq. Finally, the size of the raw files generated by PacBio SMRT and Oxford Nanopore are substantially larger than the ones for second-generation platforms, even for microbial genomes, what reflects the growing need for computational and storage resources in the context of NGS.

Fifteen years since the Human Genome Sequencing Consortium released the first draft, and 10 years after Roche released its 454 platform, DNA sequencing has undergone many changes, and many technologies have been released. Furthermore, it is possible that, in the near future, only a *de novo* assembly, without any read preprocessing or post-assembly improvement, will be enough to generate high-quality finished genomes. However, such procedures are nowadays still necessary to achieve a reliable result.

## Supplementary material

The following online material is available for this article:

Table S1 - Examples of sequenced microbial genomes for which the tools discussed in the present review were used.
