# Supplementary material for: Approaches for in silico finishing of microbial genome sequences
Source: Genet Mol Biol. 2017 Jul-Sep;40(3):553–76. doi: 10.1590/1678-4685-GMB-2016-0230 (PMC5596377; doi:10.1590/1678-4685-GMB-2016-0230)
Supplement: Supplementary file 1 [file 1415-4757-gmb-40-03-0553-Suppl01.pdf]

Genetics and Molecular Biology, 40, 3 (2017)

Supplementary material to “Approaches for in silico finishing of microbial genome sequences”

Table S1 - Examples of sequenced microbial genomes for which the tools discussed in the present review were used.

Organism	Tool	Tool Category	Sequencing Platform	Reference
Tannerella forsythia	MUMmer	Reference-based scaffolding	Illumina	(Stafford *et al.*, 2016)
Herpesvirus	ABACAS	Reference-based scaffolfing	Illumina and Sanger	(Tweedy *et al.*, 2015)
Fusarium avenaceum	ABACAS	Reference-based scaffolfing	454	(Lysøe *et al.*, 2014)
Brucella abortus	ABACAS	Reference-based scaffolfing	Illumina	(Ledwaba *et al.*, 2014)
Corynebacterium ulcerans	CONTIGuator	Reference-based scaffolfing	Ion Torrent	(Benevides *et al.*, 2015)
Bacillus pumilus	CONTIGuator	Reference-based scaffolfing	Illumina	(de Jong *et al.*, 2015)
Sacharomyces cerevisiae	CONTIGuator	Reference-based scaffolfing	Illumina	(Batista *et al.*, 2014)
Pseudomonas fluorescens	Mauve	Reference-based scaffolfing	Illumina	(Lo *et al.*, 2015)
Escherichia coli	Mauve	Reference-based scaffolfing	Illumina	(Galia *et al.*, 2015)
Pseudomonas simiae	Mauve	Reference-based scaffolfing	Illumina	(Adam *et al.*, 2015)
Bacillus thuringiensis	SIS	Reference-based scaffolfing	SOLiD and Illumina	(Varani *et al.*, 2013)
Microcystis aeruginosa	SIS	Reference-based scaffolfing	SOLiD, Ion Torrent and Illumina	(Fiore *et al.*, 2013)
Pseudomonas sp.	MeDuSa	Reference-based scaffolfing	Illumina	(Presta *et al.*, 2016)
Burkholderia cepacia	MeDuSa	Reference-based scaffolfing	Illumina	(Miller *et al.*, 2015)
Actinoplanes sp.	Ragout	Reference-based scaffolfing	Illumina	(Centeno-Leija *et al.*, 2016)
Neisseria meningitidis	Minimus	Assembly integration	Illumina and 454	(Neri *et al.*, 2014)
Cellulosimicrobium sp. MM	Minimus	Assembly integration	Illumina and 454	(Sharma *et al.*, 2014)
Roseibacterium elongatum	Minimus	Assembly integration	Illumina, 454 and Sanger	(Riedel *et al.*, 2014)
Rhizobium etli	MAIA	Assembly integration	Illumina	(Fauvart *et al.*, 2011)
Rhizobium sp. UR51a	CISA	Assembly integration	Illumina	(de Souza *et al.*, 2015)
Mycoplasma hyosynoviae	CISA	Assembly integration	Illumina	(Bumgardner *et al.*, 2015)
Xylella fastidiosa	CISA	Assembly integration	Illumina	(Giampetruzzi *et al.*, 2015)
Bacillus niacin	Mix	Assembly integration	Illumina	(Harvey and Snider, 2014)
Piscirickettsia salmonis	Mix	Assembly integration	454, Illumina and Ion Torrent	(Yañez *et al.*, 2014)
Cupriavidus sp.	Mix	Assembly integration	Illumina	(Vilo *et al.*, 2014)
Coxiella burnetii	GAM-NGS	Assembly integration	Illumina	(Walter *et al.*, 2014)
Saccharomyces cerevisiae	Zorro	Assembly integration	Illumina and 454	(Argueso *et al.*, 2009)
Vibrio campbellii	GapFiller	Gap-closing	Illumina	(Gan *et al.*, 2015)
Clostridium beijerinckii	GapFiller	Gap-closing	Illumina	(Little *et al.*, 2015)
Equid Herpesviruses	GapFiller	Gap-closing	Illumina	(Wilkie *et al.*, 2015)
Plasmodium reichenowi	IMAGE	Gap-closing	Illumina	(Otto *et al.*, 2014)
Plasmodium gaboni	IMAGE	Gap-closing	Illumina	(Otto *et al.*, 2014)

Organism	Tool	Tool Category	Sequencing Platform	Reference
Xanthomonas fagrariae	IMAGE	Gap-closing	Illumina	(Vandroemme *et al.*, 2013)
Bacillus thuringiensis	IMAGE	Gap-closing	Illumina and 454	(Iatsenko *et al.*, 2014)
Weissella ceti	FGAP	Gap-closing	Ion Torrent	(Figueiredo *et al.*, 2014)
Lactococcus lactis	FGAP	Gap-closing	Ion Torrent	(Oliveira *et al.*, 2014)
Ranavirus	iCORN	Assembly correction	Illumina	(van Beurden *et al.*, 2014)
Babesia divergens	iCORN	Assembly correction	Illumina, 454 and PacBio SMRT	(Cuesta *et al.*, 2014)
Burkholderia pseudomallei	iCORN	Assembly correction	Illumina	(Sahl *et al.*, 2013)
Klebsiella pneumonia	iCORN	Assembly correction	Illumina and 454	(Fookes *et al.*, 2013)
Ornithobacterium rhinotracheale	SEQuel	Assembly correction	Illumina and 454	(Zehr *et al.*, 2014)
Lichtheimia ramose	SEQuel	Assembly correction	Illumina and 454	(Linde *et al.*, 2014)

References

Adam Z, Chen Q, Xu R, Diange AE, Bromfield ESP and Tambong JT (2015) Draft genome sequence of *Pseudomonas simiae* Strain 2-36, an *in vitro* antagonist of *Rhizoctonia solani* and *Gaeumannomyces graminis*. Genome Announc 3:e01534-14.

Argueso JL, Carazzolle MF, Mieczkowski PA, Duarte FM, Netto OVC, Missawa SK, Galzerani F, Costa GGL, Vidal RO, Noronha MF, *et al.* (2009) Genome structure of a *Saccharomyces cerevisiae* strain widely used in bioethanol production. Genome Res 19:2258-2270.

Batista TM, Marques ETA, Franco GR and Douradinha B (2014) Draft Genome Sequence of the Probiotic Yeast *Saccharomyces cerevisiae* var. boulardii Strain ATCC MYA-796. Genome Announc 2:e01345-14.

Benevides L de J, Viana MVC, Mariano DCB, Rocha F de S, Bagano PC, Folador EL, Pereira FL, Dorella FA, Leal CAG, Carvalho AF, *et al.* (2015) Genome sequence of *Corynebacterium ulcerans* Strain FRC11. Genome Announc 3:e00112-15.

Bumgardner EA, Kittichotirat W, Bumgarner RE and Lawrence PK (2015) Comparative genomic analysis of seven *Mycoplasma hyosynoviae* strains. Microbiologyopen 4:343-359.

Centeno-Leija S, Vinuesa P, Rodríguez-Peña K, Trenado-Uribe M, Cárdenas-Conejo Y, Serrano-Posada H, Rodríguez-Sanoja R and Sánchez S (2016) Draft genome sequence of an endophytic *Actinoplanes* species, encoding uncommon trans-acyltransferase polyketide synthases. Genome Announc 4:e00164-1.

Cuesta I, González LM, Estrada K, Grande R, Zaballos A, Lobo CA, Barrera J, Sanchez-Flores A and Montero E (2014) High-quality draft genome sequence of *Babesia divergens*, the etiological agent of cattle and human abesiosis. Genome Announc 2:e01194-14.

de Jong A, van Heel AJ, Montalban-Lopez M, Krawczyk AO, Berendsen EM, Wells-Bennik M and Kuipers OP (2015) Draft genome sequences of five spore-forming food isolates of *Bacillus pumilus*. Genome Announc 3:e01539-14.

de Souza R, Sant'Anna FH, Ambrosini A, Tadra-Sfeir M, Faoro H, Pedrosa FO, Souza EM and Passaglia LMP (2015) Genome of *Rhizobium* sp.

UR51a, isolated from rice cropped in southern Brazilian fields. Genome Announc 3:e00249-15.

Fauvart M, Sánchez-Rodríguez A, Beullens S, Marchal K and Michiels J (2011) Genome sequence of *Rhizobium etli* CNPAF512, a nitrogen-fixing symbiont isolated from bean root nodules in Brazil. J Bacteriol 193:3158-3159.

Figueiredo HCP, Leal CAG, Dorella FA, Carvalho AF, Soares SC, Pereira FL and Azevedo VAC (2014) Complete genome sequences of fish *Pathogenic Weissella ceti* Strains WS74 and WS105. Genome Announc 2:e01014-14.

Fiore MF, Alvarenga DO, Varani AM, Hoff-Risseti C, Crespim E, Ramos RTJ, Silva A, Schaker PDC, Heck K, Rigonato J, *et al.* (2013) Draft genome sequence of the Brazilian toxic bloom-forming Cyanobacterium *Microcystis aeruginosa* Strain SPC777. Genome Announc 1:e00547-13.

Fookes M, Yu J, De Majumdar S, Thomson N and Schneiders T (2013) Genome sequence of *Klebsiella pneumoniae* Ecl8, a reference strain for targeted genetic manipulation. Genome Announc 1:e00027-12.

Galia W, Mariani-Kurkdjian P, Loukiadis E, Blanquet-Diot S, Leriche F, Brugère H, Shima A, Oswald E, Cournoyer B and Thevenot-Sergentet D (2015) Genome sequence and annotation of a human infection isolate of *Escherichia coli* O26:H11 involved in a raw milk cheese outbreak. Genome Announc 3:e01568-14.

Gan HY, Noor MEM, Saari NA, Musa N, Mustapha B, Usup G and Danish-Daniel M (2015) Genome sequence of *Vibrio campbellii* Strain UMTGB204, a marine bacterium isolated from a green barrel tunicate. Genome Announc 3:e00210-15.

Giampetruzzi A, Chiumenti M, Saponari M, Donvito G, Italiano A, Loconsole G, Boscia D, Cariddi C, Martelli GP and Saldarelli P (2015) Draft genome sequence of the *Xylella fastidiosa* CoDiRO Strain. Genome Announc 3:e01538-14.

Harvey ZH and Snider MJ (2014) Draft genome sequence of the nicotinate-metabolizing soil bacterium *Bacillus niacini* DSM 2923. Genome Announc 2:e01251-14.

Iatsenko I, Corton C, Pickard DJ, Dougan G and Sommer RJ (2014) Draft genome sequence of highly nematicidal *Bacillus thuringiensis* DB27. Genome Announc 2:e00101-14.

Ledwaba B, Mafofo J and van Heerden H (2014) Genome sequences of *Brucella abortus* and *Brucella suis* strains isolated from bovine in Zimbabwe. Genome Announc 2:e01063-14.

Linde J, Schwartze V, Binder U, Lass-Flörl C, Voigt K and Horn F (2014) *De novo* whole-genome sequence and genome annotation of *Lichtheimia ramosa*. Genome Announc 2:e00888-14.

Little GT, Winzer K and Minton NP (2015) Genome sequence of the solvent-producing *Clostridium beijerinckii* Strain 59B, isolated from Staffordshire garden soil. Genome Announc 2:e00108-15.

Lo R, Stanton-Cook MJ, Beatson SA, Turner MS and Bansal N (2015) Draft genome sequence of *Pseudomonas fluorescens* SRM1, an isolate from spoiled raw milk. Genome Announc 3:e00138-15.

Lysøe E, Harris LJ, Walkowiak S, Subramaniam R, Divon HH, Riiser ES, Llorens C, Gabaldón T, Kistler HC, Jonkers W, *et al.* (2014) The genome of the generalist plant pathogen *Fusarium avenaceum* is enriched with genes involved in redox, signaling and secondary metabolism. PLoS One 9:e112703.

Miller RR, Hird TJ, Tang P and Zlosnik JEA (2015) Whole-genome sequencing of three clonal clinical isolates of *B. cenocepacia* from a patient with cystic fibrosis. PLoS One 10:e0143472.

Neri A, Fazio C, Ciammaruconi A, Anselmo A, Fortunato A, Palozzi A, Vacca P, Fillo S, Lista F and Stefanelli P (2014) Draft genome sequence of C:P1.5-1,10-8:F3-6:ST-11 meningococcal clinical isolate associated with a cluster on a cruise ship. Genome Announc 2:e01263-14.

Oliveira LC, Saraiva TDL, Soares SC, Ramos RTJ, Sá PHCG, Carneiro AR, Miranda F, Freire M, Renan W, de Oliveira Júnior AFO, *et al.* (2014) Genome sequence of *Lactococcus lactis* subsp. lactis NCDO 2118, a GABA-producing strain. Genome Announc 2:e00980-14.

Otto TD, Rayner JC, Böhme U, Pain A, Spottiswoode N, Sanders M, Quail M, Ollomo B, Renaud F, Thomas AW, *et al.* (2014) Genome sequencing of chimpanzee malaria parasites reveals possible pathways of adaptation to human hosts. Nat Commun 5:4754.

Presta L, Bosi e, Fondi M, Maida I, Perrin E, Miceli E, Maggini V, Bogani P, Firenzuoli, Di Pilato V, *et al.* (2016) Draft genome sequence of *Pseudomonas* sp. EpS/L25, isolated from the medicinal plant *Echinacea purpurea* and able to synthesize antimicrobial compounds. Genome Announc 4: e00346-16.

Rice P, Longden I and Bleasby A (2000) EMBOSS: The European Molecular Biology Open Software Suite. Trends Genet 16:276-277.

Riedel T, Fiebig A, Göker M and Klenk H-P (2014) Complete genome sequence of the bacteriochlorophyll a-containing *Roseibacterium elongatum* type strain (DSM 19469(T)), a representative of the Roseobacter group isolated from Australian coast sand. Stand Genomic Sci 9:840-854.

Sahl JW, Stone JK, Gelhaus HC, Warren RL, Cruttwell CJ, Funnell SG, Keim P and Tuanyok A (2013) Genome sequence of *Burkholderia pseudomallei* NCTC 13392. Genome Announc 1:e00183-13.

Sharma A, Hira P, Shakarad M and Lal R (2014) Draft genome sequence of *Cellulosimicrobium* sp. Strain MM, isolated from arsenic-rich microbial mats of a Himalayan hot spring. Genome Announc 2:e01020-14.

Stafford GP, Chaudhuri RR, Haraszthy V, Friedrich V, Schäffer C, Ruscitto A, Honma K and Sharma A (2016) Draft genome sequences of three clinical isolates of *Tannerella forsythia* isolated from subgingival plaque from periodontitis patients in the United States. Genome Announc 4: e01286-16.

Tweedy J, Spyrou MA, Donaldson CD, Depledge D, Breuer J and Gompels UA (2015) Complete genome sequence of the Human Herpesvirus 6A Strain AJ from Africa resembles strain GS from North America. Genome Announc 3:e01498-14.

van Beurden SJ, Hughes J, Saucedo B, Rijks J, Kik M, Haenen OLM, Engelsma MY, Gröne A, Verheije MH and Wilkie G (2014) Complete genome sequence of a Common Midwife Toad virus-like Ranavirus associated with mass mortalities in wild amphibians in the Netherlands. Genome Announc 2:e01293-14.

Vandroemme J, Cottyn B, Baeyen S, De Vos P and Maes M (2013) Draft genome sequence of *Xanthomonas fragariae* reveals reductive evolution and distinct virulence-related gene content. BMC Genomics 14:829.

Varani AM, Lemos MVF, Fernandes CC, Lemos EGM, Alves ECC and Desidério JA (2013) Draft genome sequence of *Bacillus thuringiensis* var. thuringiensis Strain T01-328, a Brazilian isolate that produces a soluble pesticide protein, Cry1Ia. Genome Announc 1:e00817-13.

Vilo C, Benedik MJ, Ilori M and Dong Q (2014) Draft genome sequence of *Cupriavidus* sp. Strain SK-4, a di-ortho-substituted biphenyl-utilizing bacterium isolated from polychlorinated biphenyl-contaminated sludge. Genome Announc 2:e00474-14.

Walter MC, Öhrman C, Myrtennäs K, Sjödin A, Byström M, Larsson P, Macellaro A, Forsman M and Frangoulidis D (2014) Genome sequence of *Coxiella burnetii* strain Namibia. Stand Genomic Sci 9:22.

Wilkie GS, Kerr K, Stewart JP, Studdert MJ and Davison AJ (2015) Genome sequences of equid herpesviruses 2 and 5. Genome Announc 3:e00119-15.

Yañez AJ, Molina C, Haro RE, Sanchez P, Isla A, Mendoza J, Rojas-Herrera M, Trombert A, Silva AX, Cárcamo JG, *et al.* (2014) Draft genome sequence of virulent strain AUSTRAL-005 of *Piscirickettsia salmonis*, the etiological agent of Piscirickettsiosis. Genome Announc 2:e00990-14.

Zehr ES, Bayles DO, Boatwright WD, Tabatabai LB and Register KB (2014) Complete genome sequence of *Ornithobacterium rhinotracheale* strain ORT-UMN 88. Stand Genomic Sci 9:16.
